# Sodium-Glucose Cotransporter-2 Inhibitors Improve Heart Failure with Reduced Ejection Fraction Outcomes by Reducing Edema and Congestion

**DOI:** 10.3390/diagnostics12040989

**Published:** 2022-04-14

**Authors:** Michelle Hernandez, Ryan D. Sullivan, Mariana E. McCune, Guy L. Reed, Inna P. Gladysheva

**Affiliations:** 1 Department of Medicine, College of Medicine-Phoenix, University of Arizona, Phoenix, AZ 85004, USA; hernandez.michelle@edu.uag.mx (M.H.); ryansullivan@arizona.edu (R.D.S.); marianamccune@email.arizona.edu (M.E.M.); guyreed@arizona.edu (G.L.R.); 2 School of Medicine, Universidad Autónoma de Guadalajara, Zapopan 45129, Mexico

**Keywords:** HFrEF, edema, congestion, dilated cardiomyopathy, fluid management, endothelial dysfunction

## Abstract

Pathological sodium-water retention or edema/congestion is a primary cause of heart failure (HF) decompensation, clinical symptoms, hospitalization, reduced quality of life, and premature mortality. Sodium-glucose cotransporter-2 inhibitors (SGLT-2i) based therapies reduce hospitalization due to HF, improve functional status, quality, and duration of life in patients with HF with reduced ejection fraction (HFrEF) independently of their glycemic status. The pathophysiologic mechanisms and molecular pathways responsible for the benefits of SGLT-2i in HFrEF remain inconclusive, but SGLT-2i may help HFrEF by normalizing salt-water homeostasis to prevent clinical edema/congestion. In HFrEF, edema and congestion are related to compromised cardiac function. Edema and congestion are further aggravated by renal and pulmonary abnormalities. Treatment of HFrEF patients with SGLT-2i enhances natriuresis/diuresis, improves cardiac function, and reduces natriuretic peptide plasma levels. In this review, we summarize current clinical research studies related to outcomes of SGLT-2i treatment in HFrEF with a specific focus on their contribution to relieving or preventing edema and congestion, slowing HF progression, and decreasing the rate of rehospitalization and cardiovascular mortality.

## 1. Introduction

Heart failure with reduced ejection fraction (HFrEF) is a complex and progressive clinical syndrome that results from structural or functional impairments of cardiac function (with left ventricular ejection fraction, LVEF ≤ 40%); it is typically associated with elevated natriuretic peptide (NP) levels and objective evidence of pulmonary or systemic edema or congestion [1,2,3]. Major clinical manifestations of HFrEF include dyspnea, fatigue and, malaise that linked to pulmonary and/or splanchnic congestion and/or peripheral edema [1,2,3,4,5,6,7,8,9,10]. HFrEF is a progressive disease ranging from pre-symptomatic stages A (at-risk for HF), B (pre-HF: decline in systolic function); to symptomatic stages C (true HF) and D (advanced HF) [1,2,3,6,10,11].

Despite progress in management, symptomatic HFrEF remains an irreversible condition that leads inexorably to a poor quality of life, disability and nearly 50% mortality within five years of diagnosis; mechanical circulatory support or heart transplantation being the only definitive curative measures [1,2,3].

In addition to regulating the neurohumoral system (sympathetic nervous system, SNS; renin-angiotensin-aldosterone system, RAAS; and natriuretic peptides, NPs) [12,13], recent advances in the pharmacological management of HFrEF include sodium-glucose cotransporter-2 inhibitors (SGLT-2i: canagliflozin, dapagliflozin, empagliflozin) [1,2,3,14,15]. SGLT-2i are a new class of hypoglycemic agents that were FDA-approved for the management of New York Heart Association (NYHA) class II-IV HFrEF, with or without type 2 diabetes mellitus (T2DM) [15]. SGLT-2i function by inhibiting the reabsorption of sodium (Na^+^) and glucose in the proximal convoluted tubules of the kidney by SGLT2, thereby increasing urinary excretion of sodium and glucose in a 1:1 ratio and subsequently extracellular water [16,17]. Data from multiple clinical trials including post-hoc, meta and cross-trial analyses, showed that HFrEF patients receiving guideline-directed pharmacological therapy with SGLT-2i had improved cardiovascular outcomes with a significant reduction in the combined risk of cardiovascular death or HF-related hospitalization (HHF) and renal outcomes regardless of T2DM status [18,19,20,21,22,23,24,25,26,27,28,29,30,31,32,33,34,35]. These improvements were independent from co-administration of guideline-directed HF therapy [32,36]. The glucosuria and reduced glycemic levels are insufficient to explain the overwhelming benefits of SGLT-2i on cardiovascular outcomes, HHF and survival benefits in HFrEF. The pathophysiologic mechanisms and molecular pathways underlying the benefits of SGLT-2i in HFrEF are complex and have yet to be elucidated [15,16,17,37,38,39,40,41,42,43,44,45,46,47,48].

Symptomatic HF is characterized in part by excessive sodium and fluid retention in the interstitial space (interstitium or ‘third’ space) leading to clinically evident edema/congestion in the lungs (pulmonary edema), or the thoracic cavity (pleural effusion), the abdomen (ascites) and/or dependent extremities (peripheral edema). These clinical manifestations of fluid overload are the primary cause of patient HHF and are associated with significant morbidity and premature mortality [6,7,8,9,49,50,51,52,53,54,55,56,57,58,59]. The primary goals of HF management include maintaining normal fluid homeostasis by managing sodium intake, use of HF medication(s) and pharmacological treatment of edema [59].

The aim of this review is to analyze the existing clinical data with the major focus on the potential of SGLT-2i in modulating or preventing fluid retention in patients at risk or active symptomatic HFrEF. Although edema/congestion is associated with poor HF outcomes, clinical diagnosis of edema is often limited to subjective patient symptoms and clinical signs from physical exam. Objective diagnostic imaging, laboratory tools and algorithms for assessment and early identification of edema are limited [8,9,51,56,57,59,60,61,62,63,64,65,66,67]. Limitations in the quantitative assessment of edema/congestion have hampered the evaluation of direct SGLT-2i edema-related outcomes in clinical studies and clinical trials.

## 2. Impact of SGLT-2i on Diuretic, Natriuretic and Renal Hemodynamic Outcomes in HFrEF: Focus on Edema/Congestion

The exact mechanism(s) by which SGLT-2i exert beneficial effects on HFrEF outcomes has yet to be elucidated. Currently there are numerous theories and hypothesized mechanisms focusing on cardioprotective benefits of SGLT-2i [15,16,17,37,38,39,40,41,42,43,44,45,46,47,48,68]. However, these theories when applied solely, are insufficient to explain the reduction of HHF rate and mortality by SGLT-2i-based therapies in patients with HFrEF.

**Figure 1:**
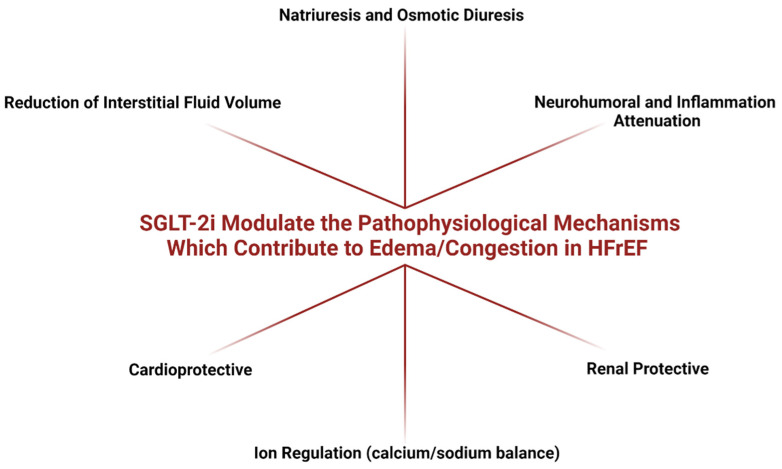
Summary of Mechanisms Contributing to Outcomes of SGLT-2i on Edema/Congestion in HFrEF [15,16,17,37,38,39,40,41,42,43,44,45,46,47,48,68,69].

HF decompensation events related to edema/congestion are the primary cause of the HHF and re-hospitalization. SGLT-2i may reduce HHF rate and benefit patients with HFrEF by concomitant beneficial modulation of pathologically deregulated mechanisms causing an imbalance of salt-water homeostasis manifesting as clinically evident edema/congestion. The mechanisms contributing to edema/congestion attenuation in HFrEF by treatment with SGLT-2i [15,16,17,37,38,39,40,41,42,43,44,45,46,47,48,68,69] are summarized on Figure 1.

### 2.1. Impact of SGLT-2i on Natriuresis and Osmotic Diuresis

SGLT-2i may benefit patients with HFrEF by promotion of natriuresis-suppression of sodium reabsorption in the kidney, which is pathologically increased in HF causing deregulation of salt-water homeostasis manifesting as clinically evident edema, and associated diuresis leading to decongestion.

Blockade of the SGLT-2 in the proximal convoluted tubule inhibits the reabsorption of 30–50% of filtered glucose. This effect is accompanied by a significant increase in urinary sodium excretion, leading to a potent combined osmotic diuretic and natriuretic effect and an ensuing favorable reduction in plasma volume, blood pressure, and interstitial fluid volume [28,29,70]. Treatment with dapagliflozin has been associated with a reduction in mean 24-h blood pressure measurements, as well as a greater reduction in body weight and plasma volume compared to hydrochlorothiazide [71]. The beneficial impact of SGLT-2i on these parameters can be attributed to the immediate diuretic action and fluid loss that occurs because of increased urinary sodium and glucose excretion. Prolonged inhibition of SGLT-2 also favorably affects sodium excretion while concurrent activation of compensatory mechanisms, such as increased renin and vasopressin secretion, restores extracellular volume homeostasis, thereby preventing the deleterious effects of excessive diuresis or dehydration [72,73].

The impressive benefits of SGLT-2i on HF outcomes may not be explained by their diuretic effects alone, since other diuretics have not been associated with reduction in HHF and death [44,74]. In fact, prolonged use of loop diuretics in the setting of acute decompensated HF has been linked to worsening outcomes and increased mortality. This is most likely due to the risk of hypotension, electrolyte imbalance, and reduced renal perfusion if inadequately titrated. Reduced arterial filling can further activate RAAS, leading to greater sodium and water retention which can worsen peripheral and pulmonary edema [39,75]. In contrast, treatment with SGLT-2i may control electrolyte balance and renal perfusion that delay or prevent renal hyperfiltration.

### 2.2. SGLT-2i Reduced Pathological HF-Related Intestinal Fluid Volume Rather Than Blood Plasma Volume

The DAPA-HF trial showed that treatment with dapagliflozin was associated with a larger increase in electrolyte free water clearance compared to a traditional loop diuretic. A mathematical integrated cardiorenal modeling analysis showed that SGLT-2i may generate Na^+^-free water clearance and its diuretic mechanism is distinct from other Na^+^-driven diuretic classes. Therefore, it was hypothesized that SGLT-2i reduces pathological HF interstitial fluid volume to a greater extent than blood plasma volume [39,76], which is in contrast to other diuretics. This difference may be mediated by peripheral sequestration of osmotically inactive sodium. It has been shown that sodium can be stored in the skin and other tissues, likely by binding with negatively charged proteoglycans [17]. When excess water relative to sodium is removed and sodium concentration within the interstitial compartment rises, peripheral tissues can sequester sodium [39]. This peripheral sequestration of sodium reduces the water volume needed to maintain the equilibrium of sodium concentration between the interstitial and intravascular compartments. This coupled with low cardiac output associated with HFrEF leads to a maladaptive neurohumoral response. If SGLT-2i relieves fluid accumulation in HF by reducing congestion in the interstitial space, arterial filling and organ perfusion may remain intact. Therefore, SGLT-2i may more efficiently impact HF edema/congestion without the deleterious effects from other types of diuretics.

### 2.3. Impact of SGLT-2i on Renal Function

Renal and cardiovascular hemodynamics are highly interdependent. In primary cardiovascular dysfunction, the heart is unable to adequately pump blood into the systemic circulation, causing prerenal hypoperfusion [77]. The reduced total renal blood flow and glomerular filtration rate (GFR) contribute to increased sodium retention and to edema formation in patients with congestive HF [8,78,79,80]. Beneficial renal hemodynamic effects of SGLT-2i observed in clinical trials in patients with and without diabetes likely support glycemic-independent nature of such improvement.

SGLT-2i can potentially mitigate the fluid retention that occurs as a result of inadequate renal blood flow. Treatment with SGLT-2i directly affects the physiology of tubuloglomerular feedback. Blockade of SGLT-2-mediated reabsorption of sodium and glucose leads to increased sodium delivery to the distal tubule [81]. Increased sodium concentration activates the juxtaglomerular apparatus and subsequent vasoconstriction of the afferent arteriole. This lowers glomerular hydrostatic pressure and decreases the GFR. Lowering the GFR at the start of treatment with SGLT-2i (GFR normalizes soon after) delays the onset of glomerular hyperfiltration that progressively leads to diabetic nephropathy. Several cardiovascular outcome trials, such as CREDENCE and DAPA-HF, reported a significant reduction in the rate of hyperfiltration at the onset of treatment as well as a long-term reduction in the overall decline of GFR [20,24,81].

Also, it was proposed that SGLT-2i could protect against renal failure by attenuating latent renal congestion by diminishing excessive sodium and water from the interstitial space of the kidney parenchyma. This mechanism might be beneficial even at asymptomatic HF [82].

### 2.4. Effects of SGLT-2i on Neurohumoral Activation

The pathophysiology of HFrEF involves prolonged activation of the SNS and classical RAAS, compromised angiotensin converting enzyme 2-angiotensin (1–7) axis of RAAS, and impairment of NP system and nitric oxide (NO) pathway associated with accumulation of sodium and body fluid, blood pressure and cardiac remodeling [13,65,83,84,85,86,87,88,89,90]. Reduced cardiac output causes persistent sympathetic activation and a maladaptive cycle that perpetuates fluid retention and edema. There exists evidence to suggest that SGLT-2i may modulate this deleterious sympathetic response. Several clinical trials have shown that treatment with SGLT-2i was not associated with a reflex increase in heart rate in response to blood pressure reduction [48]. This can be considered a marker of sympathetic suppression. Similar findings have been shown in experimental animal studies. Dampening of neurohormonal activation was seen in a group of empagliflozin-treated, nondiabetic pigs with HF. This group had lower plasma levels of norepinephrine catabolites [91].

The diuretic, natriuretic and cardiac hemodynamic outcomes of SGLT-2i associated with reduced congestion in HFrEF patients with and without T2DM might potentially lead to classical RAAS overactivation. However, the data on association between chronic treatment with SGLT-2i and classical RAAS systemic and renal activation are complicated and mostly limited to the patients with T2DM and translational models of T2DM [92].

## 3. Overview of SGLT-2i Treatment Outcomes Contributing to Edema/Congestion Modulation in HFrEF-Related Clinical Trials

The overall results of the randomized clinical trials strongly support a role for SGLT-2i in the treatment of HFrEF patients independently of their glycemic status and might suggest its effects on maintaining normal fluid homeostasis. Results of HF-associated clinical trials demonstrated benefits on cardiovascular outcomes, renal function, and plasma ANP/BNP and NT-pro-BNP levels with a significant reduction in mortality and HHF and overall quality of life.

### 3.1. Impact of SGLT-2i on Death and Hospitalization Rates

Reductions in premature mortality and HHF rates are the primarily endpoints in the HFrEF-associated clinical trials. In randomized controlled trials, SGLT-2i added to guideline-directed pharmacological therapy reduced HHF or cardiovascular-related mortality in HFrEF. As HF-related edema is strongly associated with HHF and mortality [5,6,7,49,50,51,52,54,57,58,59,60,93,94,95] a potential SGLT-2i impact on edema attenuation may contribute to the reduction in HHF and premature mortality. The major outcome measures in primary HFrEF clinical trials are summarized in Table 1.

SGLT-2i have become the subject of investigation in several cardiovascular outcome trials within the past six years. Trial data assessing cardiovascular risk in patients with T2DM treated with SGLT-2i has shown possible off target benefits for cardiovascular disease and HF. The EMPA-REG OUTCOME trial demonstrated a significant reduction in the rate of major adverse cardiac events (MACE) in patients with T2DM and established atherosclerotic cardiovascular disease (ASCVD) treated with empagliflozin [18]. The CANVAS trial yielded similar results in the same target population of patient with T2DM and ASCVD treated with canagliflozin [19]. These trials, as well as DECLARE-TIMI and CREDENCE, also demonstrated a significant reduction in the rate HHF [18,19,20,97]. However, these studies primarily included diabetic patients without evidence of pre-existing HF at baseline. The DAPA-HF (Dapagliflozin and Prevention of Adverse outcome in Heart Failure) trial was a cardiovascular outcome trial designed to assess the effect of SGLT-2i in patients with pre-existing HFrEF, with or without T2DM. The primary outcome of a composite of a first episode of worsening HF (HHF or an urgent visit resulting in IV therapy for HF) or cardiovascular death occurred in 16.3% of patients in the dapagliflozin group versus 21.2% in the placebo group [24]. This finding was significant across all prespecified subgroups, including those with and without T2DM. In this trial, patients experienced less symptoms of HF in the dapagliflozin arm compared to conventional therapy. This was evidenced by improvement in the Kansas City Cardiomyopathy Questionnaire (KCCQ) score. This finding was also homogenous across prespecified subgroups. The EMPEROR-Reduced trial (assessing outcome of empagliflozin in HFrEF) evaluated the same target population as DAPA-HF, however it included patients with markedly reduced EF and elevated NPs [27]. The primary outcome was a composite of HHF or cardiovascular death. The overall combined risk was 25% lower in the empagliflozin group than in the placebo group [27]. Patients in the empagliflozin group were also more likely to experience an improvement in NYHA functional class compared to the placebo group. In the pilot EMPA-RESPONSE-AHF, treatment with empagliflozin reduced a combined endpoint of worsening HF, HHF or death for 60 days in patients with acute HF [29]. Dapagliflozin reduced the risk of total (first and repeat) HHF and cardiovascular death [98]. A post hoc analysis of DAPA-HF showed that in patients with dapagliflozin reduced the risk of sudden death when added to conventional therapy [99]. Dapagliflozin reduced the risk of worsening HF, cardiovascular death, and all-cause death irrespective of sex [100]. Thus, data from the multiple clinical trials show that in HFrEF SGLT-2i improves cardiovascular outcomes with a significant reduction in mortality and HHF regardless of T2DM status [14,18,19,20,21,22,23,24,25,26,27,28,29,30,31,32,33,34,35,47,97]. A meta-analysis investigating the overall effect of SGLT-2i on cardiovascular outcomes in patients with HF concluded that they significantly reduced the risk of cardiovascular death and HHF by 23%. SGLT-2i were robustly effective in HFrEF subgroup regardless of T2DM and tended to be effective in HFpEF [101].

In summary, randomized clinical trials support the hypothesis that SGLT-2i provides cardiovascular benefits and a reduction in HHF rates via an unknown mechanism in addition to those affecting blood glucose regulation and might suggest that SGLT-2i benefits patients with HFrEF with or without T2DM in part through attenuation of edema/congestion.

### 3.2. Impact of SGLT-2i on Cardiac Remodeling and Metabolism

The EMPA-TROPISM double-blind, placebo-controlled clinical trial evaluated the effects of empagliflozin on cardiac remodeling in nondiabetic patients with HFrEF [96]. Treatment with empagliflozin was associated with significant reductions in LV end systolic volume and end diastolic volume as well as increased EF and decreased LV mass [96].

A secondary analysis of the comparative cardiac MRI data at baseline and after 6 months of nondiabetic HFrEF patients enrolled in the EMPA-TROPISM clinical trial revealed that empagliflozin significantly improved adiposity (epicardial adipose tissue), interstitial myocardial fibrosis, aortic stiffness [102].

MRI-determined LV end-systolic volume and LV end-diastolic volume index were significantly reduced in another randomized, double-blind, placebo-controlled trial (SUGAR-DM-HF) with HFrEF patients (NYHA functional class II to IV) with prediabetes/diabetes treated with empagliflozin [103].

The EMPA-VISION clinical trial (double-blind, randomized, placebo-controlled) was designed to assess the effects of empagliflozin treatment on cardiac energy metabolism in HFrEF patients with or without T2DM using longitudinal MRI [104]. Additional studies are underway to explore the potential metabolic alterations from SGLT-2i therapy for HFrEF. Important to note, cardiac cachexia and sarcopenia which result from a negative metabolic state of advanced HFrEF, may be an additional source of extracellular water via hydrolysis (See 3.6 for Impacts of SGLT-2i on Fluid Retention).

### 3.3. Impact of SGLT-2i on Plasma Biomarkers

Plasma levels of ANP/BNP and their NT-pro-forms are strongly associated with HFrEF decompensation related to clinical symptoms from fluid and salt retention and play crucial role in the clinical assessment of decompensation in patients with HF [5,6,105,106,107].

The impact of the SGLT-2i on a broad range of plasma biomarkers in clinical and pre-clinical HFrEF was comprehensively reviewed [108]. Here, we briefly summarize the influence of SGLT-2i on plasma levels of NPs and inflammatory markers that might indicate congestion status in patients with HFrEF in randomized clinical trials.

In the DEFINE-HF clinical trial, HFrEF patients (NYHA functional class II–III, eGFR ≥ 30 mL/min/1.73 m^2^, and elevated NT-proBNP) receiving dapagliflozin over 12 weeks did not show difference n mean NT-proBNP, however, there were increases in the proportion of patients with clinically meaningful improvements in KCCQ score or NPs [25]. In DAPA-HF trial treatment of HFrEF patients (NYHA functional class II–IV, increased NT-pro-BNP) with dapagliflozin for 8 months caused significant reduction in NT-proBNP levels (by 300 pg/mL) vs. placebo, which was consistent with a reduction of the risk of HF progression, death and improved HF symptoms [109]. In the EMPEROR-Reduced trial (HFrEF patients with elevated levels of BNP), patients treated with empagliflozin experienced greater reductions in NT-proBNP concentrations compared to placebo. There was also a reduced risk of adverse HF outcomes regardless of baseline NT-proBNP levels [27]. Treatment with empagliflozin significantly reduced plasma ANP/BNP levels when assessed after 1, 3, 6 and 12 months vs. baseline in small randomized study of Japanese patients with chronic HFrEF and T2DM [110]. It is important to note that assessments of the NT-pro-BNP level may be affected by chronic kidney dysfunction (eGFR ≤ 60 mL/min/1.73 m^2^) [84,111,112], and increased body mass index [88,112].

Chronic sustained inflammation promotes pathological cardiac remodeling, LV dysfunction, pleural/pulmonary/systemic edema in HFrEF [113,114,115]. Therefore, inflammatory plasma biomarkers might indirectly reflect the congestion status in HFrEF patients. Proteomic analysis revealed that treatment of nondiabetic HFrEF patients with empagliflozin vs. placebo (EMPA-TROPISM clinical trial) was associated with a significant reduction in inflammatory biomarkers [102]. Therapy with SLT-2i decreased plasma levels of inflammatory markers such as tumor necrosis factor-1, fibronectin, and matrix metalloproteinase 7 in patients randomized to canagliflozin vs. glimepiride [116]. These results suggest that SGLT-2i contributes to the suppression of inflammation-related molecular processes and may attenuate associated endothelial dysfunction as supported by preclinical studies [117,118,119], and therefore prevent vascular leakage which contributes to edema/congestion.

### 3.4. Impact of SGLT-2i on Renal Function

Chronic renal dysfunction (eGFR ≤ 60 mL/min/1.73 m^2^) accelerates HFrEF decompensation and mortality [120,121,122]. The DAPA-HF trial assessed the safety and efficacy of dapagliflozin in patients with HFrEF with or without T2DM. Treatment with dapagliflozin slowed the rate of decline in eGFR. The benefits of dapagliflozin on morbidity and mortality were not affected by baseline kidney function [123]. The rate of decline in eGFR was slower in the empagliflozin group than in the placebo group in the EMPEROR-Reduced trial [27].

The experimental trials with canagliflozin (CREDENCE) and prespecified subgroup meta-analyses from DAPA-HF (dapagliflozin) and EMPEROR-Reduced (empagliflozin) have demonstrated that SGLT-2i has beneficial effects on renal outcomes in patients with HFrEF regardless of T2DM and chronic kidney disease (CKD) status [32,41,84,85]. Treatment with canagliflozin was associated with 30% reduction in the primary composite outcome of end-stage kidney disease (dialysis, transplantation, or sustained eGFR of <15 mL/min/1.73 m^2^) [20]. A secondary composite outcome of ESRD, doubling of serum creatinine, and renal death was reduced by 34% [20]. These improved renal outcomes were observed in addition to improved cardiovascular outcomes. Treatment with canagliflozin was associated with a 20% decrease in the risk of myocardial infarction (MI), stroke, and cardiovascular death. HHF was reduced by 39% [20]. These findings were observed in conjunction with only a slight reduction in Hemoglobin A1c, which suggests that the mechanisms of the observed benefit are independent of the glucose lowering effect of SGLT-2i.

### 3.5. Impact of SGLT-2i on Functional Status and Quality of Life

In many clinical trials (DEFINE-HF, DAPA-HF, EMPEROR-REDUCED, EMPA-TROPISM, and SOLOIST-WHF), HFrEF patients treated with SGLT-2i experienced reduced clinical symptoms of HF compared to patients treated with only conventional therapy, as was evidenced by improvement in the KCCQ score from baseline [124]. The EMPA-TROPISM clinical trial evaluated the effects of empagliflozin on functional capacity, and quality of life nondiabetic patients with HFrEF [96]. HFrEF patient functional status was significantly improved in the empagliflozin sub-group vs. placebo group, evidenced by enhanced oxygen consumption and improvement in a 6-min walk test. Patients in the empagliflozin treatment sub-group also reported a lower symptom burden and improved quality of life (21 +/− 18 vs. 2 +/− 15; *p* < 0.001) on the KCCQ [96]. A secondary analysis of the EMPEROR-Reduced trial found that empagliflozin significantly improved patient health status as assessed by the KCCQ-CSS by 1.5 to 2.0 points. Empagliflozin led to more 5-point, 10-point, and 15-point improvements in and fewer deteriorations in KCCQ-CSS at 3 months compared to placebo [125]. The improved functional status assessed by KCCQ was also reported for HFrEF patients treated with dapagliflozin vs. placebo in DEFINE-HF [25] and DAPA-HF trials [30,109]. Treatment of HFrEF patients with dapagliflozin improved clinical symptoms, physical function, and health-related quality of life regardless of sex [100]. Using a new type of study design, the CHIEF-HF trail (randomized, double-blind, controlled), focused on patient-centered outcomes and conducted in a completely remote fashion, showed canagliflozin significantly improved patient symptom burden after 12 weeks of treatment (KCCQ Total Symptom Score improvement by 4.3 points), regardless of EF or T2DM status [126]. Collectively, these data emphasize substantial benefits of SGLT-2i in improvement of HF symptoms, function and quality of life in HFrEF patients.

### 3.6. Direct Impact of SGLT-2i on Fluid Retention

One method commonly used to quantify the extent of peripheral edema is fluid weight gain. Several of the HF-associated clinical trials reported an overall decrease in weight and waist circumference in the patients treated with an SGLT-2i [106]. Meta-analysis of eight randomized-controlled trials (a combined cohort of 5233 HF patients without T2DM) reported 20% relative risk reduction in cardiovascular and HHF, associated with a reduction in body weight (−1.21 kg, *p* < 0.001), body mass index (−0.47 kg/m^2^, *p* < 0.001) in patients treated with SGLT-2i vs. those without treatment [127]. The post-hoc analysis of the SONAR trial (patients with T2DM and CKD), show that six-weeks treatment SGLT-2i added to an endothelin receptor antagonist decreased body weight, a surrogate for fluid retention [128].

Since major HF symptoms are associated with fluid retention and vascular congestion, SGLT-2i must significantly improve the volume status of patients with HF. An ongoing clinical trial called EMPULSE is currently investigating the effects of empagliflozin on all-cause mortality and HHF for acute HF with signs of dyspnea, fluid overload, and elevated NPs [107]. This trial will be assessing the clinical benefit and safety of empagliflozin in this population. The results will shed light on whether SGLT-2i provides any benefits in patients with acute symptomatic HF.

## 4. Discussion

Two independent clinical trials, DAPA-HF and EMPEROR-Reduced, showed that SGLT-2i (dapagliflozin and empagliflozin), reduce HHF and all-cause cardiovascular death, and improved renal outcomes in HFrEF patients with or without diabetes independently from co-administration of OMT. These significant benefits have been associated with improving functional status and quality of life. The pathophysiologic mechanisms and molecular pathways underlying the benefits of SGLT-2i in HFrEF are complex and remain incompletely understood.

The HHF and re-hospitalization rates reflect HF decompensation events related to clinical symptoms caused by edema/congestion. Thus, reducing HHF/re-hospitalization is directly related to the suppression/prevention of edema/congestion exacerbation. In patients with HFrEF, edema/congestion develops, as a pathophysiologic outcome, under control of interdependent functional crosstalk between dynamic cardiac function and remodeling, chronic inflammation, endothelial dysfunction, changes in peripheral vasculature system, and pathological neurohormonal activation of SNS-RAAS and impairment of NPs and NO-related mechanisms. Comorbidities with renal or pulmonary abnormalities aggravate these pathologies. Treatment of HFrEF patients (with or without T2DM) with SGLT-2i leads to diuresis/natriuresis stimulation (reduced volume overload), improvement of overall cardiac function (improved cardiac output), and reduction (significant or mild) of NP plasma levels. The impact of the SGLT-2i on the classical RAAS overactivation and impairment of NP/NO mechanisms in HFrEF remains to be determined.

**Figure 2:**
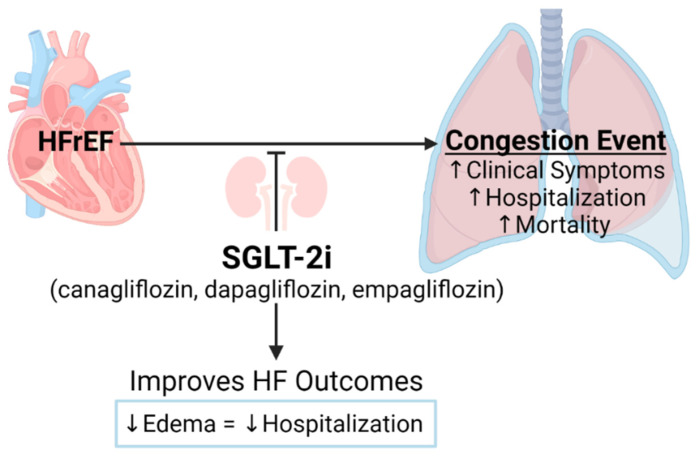
Schematic presentation of SGLT-2i Contribution to Attenuation of Edema/Congestion in HFrEF. Created with BioRender.com.

In contrast with other diuretics, treatment with SGLT-2i likely controls electrolyte balance and renal perfusion. A mathematic integrated cardiorenal modeling analysis of the DAPA-HF clinical trial predicts that SGLT-2i relives HF-related edema/congestion by reducing pathologically elevated interstitial fluid volume without significantly reducing blood plasma volume (i.e., normalizing blood volume homeostasis—or reducing volume overload stress on the heart). The ability of SGLT-2i to suppress chronic inflammation and, eventually, attenuate endothelial dysfunction suggests that SGLT-2i may control/prevent fluid leakage from the vascular compartment to the interstitial space and prevent edema development. The summary of SGLT-2i outcomes in HFrEF associated with edema/congestion repressing shown in the Figure 2.

The prevention and detection of pathological fluid is important for HF outcomes. SGLT-2i appear to prevent and delay HF progression, but additional research is needed and ongoing.

## 5. Conclusions

Late stage HF is characterized by the generation of edema/congestion leading to clinical symptoms and hospitalization. Clinical HFrEF studies summarized in this review suggest that SGLT-2i treatment may attenuate the pathological salt-water retention; however, additional studies should be designed to investigate HFrEF edema/congestion as a primary outcome.

## Figures and Tables

**Table 1:** SGLT-2i treatment outcome in primary HFrEF clinical trials.

HF Clinical Trial	Study Population Inclusion Criteria	Major Outcome Measures	Summary
**DAPA-HF** Dapagliflozin: 10 mg or 5 mg tablets given once daily/up to 27.8 months. McMurray et al., 2019 [24]	HFrEF (LVEF ≤ 40%), with/without T2DM. Sample size—4744 Male/female, ≥18 years Symptomatic HFrEF (NYHA functional class II–IV), for ≥2 months LVEF ≤ 40% Elevated NT-proBNP Patients should receive background OMT for HFrEF according to locally recognized guidelines eGFR ≥ 30 mL/min/1.73 m^2^ at enrolment	Dapagliflozin vs. pacebo groups: Composite of a first episode of worsening HF (hospitalization or an urgent visit resulting in IV therapy for HF) or CV death-event rate of 16.3% vs. 21.2%; Composite of HHF or CV death-lower; (HR = 0.75, 0.65–0.85) Total number of hospitalizations for HF and CV death-fewer (16.1% vs. 20.9%)	Dapagliflozin reduced HHF and CV death
**EMPEROR-REDUCED** Empagliflozin: 10 mg/once daily/up to 1040 days Packer et al., 2020 [27]	Chronic HFrEF (LVEF ≤ 40%), irrespective of diabetes status. Sample size—3730 Male/female, ≥18 years EF ≥ 36% to ≤40%: NT-proBNP ≥ 500 pg/mL or patients without AF and NT-proBNP ≥ 5000 pg/mL for patients with AF EF ≥ 31% to ≤35%: NT-proBNP ≥1000 pg/mL for patients without AF and NT-proBNP ≥ 2000 pg/mL for patients with AF EF ≤ 30%: NT-proBNP ≥ 600 pg/mL for patients without AF and NT-proBNP ≥ 1200 pg/mL for patients with AF EF ≤ 40% and hospitalization for HF in the past 12 months: NT-proBNP ≥ 600 pg/mL for patients without AF and NT-proBNP ≥ 1200 pg/mL for patients with AF	Empagliflozin vs. placebo group: Reduced hospitalization for worsening HF or CV death; Overall combined risk was 25% lower in the empagliflozin group The occurrence of all adjudicated hospitalizations for HF (first and recurrent events)—31% lower; Rate of decline in the eGFR was slower	Empagliflozin reduced HHF and CV death; preserved renal function
**EMPA-TROPISM** Empagliflozin: 10mg/once daily/6 months Santos-Gallegos et al., 2021 [96]	HFrEF Sample Size—84 Male/female, ≥18 years Diagnosis of Heart failure (NYHA II to III) LVEF < 50% on echocardiography cMRI in the previous 6 months Have stable symptoms and therapy for HF within the last 3 months.	Empagliflozin vs. placebo group from baseline to 6 months: LV end-systolic volume: 26.6 mL vs. −0.5 mL (*p* < 0.001); LV end-diastolic volume: 25.1 vs. −1.5 mL (*p* < 0.001); LVEF: 6.0% vs. −0.1% (*p* < 0.001); LV mass: −17.8 g/m^2^ vs. 4.1 g/m^2^ (*p* < 0.001); Peak VO2: 1.1 mL/kg/min vs. −0.5 mL/kg/min (*p* = 0.017); 6-min walk test: 82 vs. −35 min (*p* < 0.001).	Empagliflozin improved cardiac function (suggesting cardiac pressure overload improvement) and patient exercise capacity
**EMPA-RESPONSE-AHF** Empagliflozin: 10 mg/daily/30 days Damman et al., 2020 [29]	Acute HF, Congestive HF with decompensation Sample size—80 Male/female, ≥18 years Hospitalized for AHF: - Dyspnea at rest - Signs of congestion, such as edema, rales, and/or congestion on chest radiograph - BNP ≥ 350 pg/mL or NT-proBNP ≥ 1400 pg/mL (Patients with AF: BNP ≥ 500 pg/mL or NT-proBNP ≥ 2000 pg/mL) Treated with loop diuretics Able to be randomized within 24 h Able and willing to provide freely given written informed consent eGFR (CKD-EPI) ≥ 30 mL/min/1.73 m^2^	Empagliflozin vs. placebo group: No difference was observed in VAS dyspnea score, diuretic response, length of stay, or change in NT-proBNP; Reduced a combined endpoint of in-hospital worsening HF, rehospitalization for HF or death at 60 days compared with placebo [4 (10%) vs. 13 (33%); *p* = 0.014]; Urinary output significantly greater [difference 3449 (95% confidence interval 578–6321) mL; *p* < 0.01]; No adverse effects on blood pressure or renal function.	Empagliflozin reduced HHF; acute setting and small sample size limited results

Dapagliflozin and Prevention of Adverse-outcomes in Heart Failure (DAPA-HF); Empagliflozin Outcome in Chronic Heart Failure with Reduced Ejection Fraction (EMPEROR-REDUCED); Are the “Cardiac Benefits” of Empagliflozin Independent of Its Hypoglycemic Activity? (EMPA-TROPISM); Effects of Empagliflozin on Clinical Outcomes in Patients with Acute Decompensated HF (EMPA-RESPONSE-AHF); Type 2 diabetes mellitus (T2DM); Atherosclerotic cardiovascular disease (ASCVD); Myocardial infarction (MI); Cardiovascular (CV); CV disease (CVD); Hospitalization for heart failure (HHF); Heart failure (HF); Left ventricular ejection fraction (LVEF); Heart failure with reduced ejection fraction (HFrEF); Left ventricular (LV); Estimated glomerular filtration rate (eGFR); End-stage renal disease (ESRD); Chronic kidney disease (CKD); Hazard ratio (HR); Atrial fibrillation (AF).

## Abbreviations

AF	Atrial Fibrillation
ANP/NT-proANP	Atrial natriuretic peptide/N-terminal-proANP
ASCVD	Atherosclerotic cardiovascular disease
BNP/NT-proBNP	Brain/B-type natriuretic peptide/N-terminal-proBNP
CKD	Chronic kidney disease
CSS	Clinical summary score
CV	Cardiovascular
EF	Ejection fraction
eGFR	Estimated glomerular filtration rate
ESRD	End-stage renal disease
HF	Heart failure
HFrEF	Heart failure with reduced ejection fraction
HFpEF	Heart failure with preserved ejection fraction
HHF	Hospitalization due to heart failure
HR	Hazard ratio
KCCQ	Kansas City Cardiomyopathy Questionnaire
LVEF	Left ventricular ejection fraction
MACE	Major adverse cardiac events
NO	Nitric oxide
NPs	Natriuretic peptides
NYHA	New York Heart Association
OMT	Optimal medical therapy
RAAS	Renin-angiotensin-aldosterone system
SGLT-2i	Sodium-glucose cotransporter-2 inhibitors
SNS	Sympathetic nervous system
T2DM/T1DM	Type 2/Type 1 diabetes mellitus
